# LDL receptor expression on T lymphocytes in old patients with Down syndrome

**DOI:** 10.1186/1742-4933-2-3

**Published:** 2005-02-10

**Authors:** Massimiliano M Corsi, Alexis E Malavazos, Daniele Passoni, Federico Licastro

**Affiliations:** 1 Institute of General Pathology, Laboratory of Clinical Pathology, Faculty of Medicine, University of Milan, Italy; 2 Department of Experimental Pathology, Section of Immunology, Faculty of Medicine, University of Bologna, Italy

## Abstract

**Background:**

In Down syndrome patients several metabolic abnormalities have been reported, some involving the lipid metabolism. The level of LDL in plasma is the major determinant of the risk of vascular disease. There appear to be no studies on the LDL receptor in Down syndrome patients.

**Methods:**

Flow cytometric methods for measuring the LDL receptor in peripheral blood mononuclear cells (PBMC) can identify patients with hypercholesterolemia. We applied this method in 19 old patients with Down syndrome and 23 healthy controls.

**Results:**

Down syndrome patients had high levels of triglycerides and low levels of HDL, and high levels of CRP. We also found a down-regulation of LDL receptor expression.

**Conclusions:**

Down syndrome patients show no increase in the frequency of cardiovascular disease. The low incidence in cardiovascular disease despite the low level of HDL, high levels of CRP and reduction of LDL receptor expression lead to the conclusion that either these are not risk factors in these patients or that other risks factors – not yet identified – are considerably lower.

## Introduction

Several studies have discussed the psychological and intellectual problems, immunological deficiencies, and early aging of Down syndrome (DS) patients. Several metabolic abnormalities have been reported, some involving the lipid metabolism [1]. Apart from some contradictory studies in the past, there are only few investigations of the cholesterol fractions in DS patients. Therefore, it must be concluded that the low prevalence of coronary artery disease in individuals with DS cannot be explained by their cholesterol fractions. Mortality statistics of these patients showed practically no deaths due to advanced atherosclerosis [2], and similarly, pathological studies have detected no increase in atherosclerosis – or even a complete absence of atherosclerotic changes [3].

In children [4] and also adolescents [5] with DS low levels of high-density lipoprotein (HDL) have been reported and recently, we have learned much about the vasoprotective HDL cholesterol [6]. Anyway DS remains a disease in which atherosclerosis is rare [7].

Measurements of LDL receptor expression are also necessary to fully characterize the functional status of the low-density lipoprotein (LDL) pathway which substantially influences LDL levels in plasma, and its discovery constituted a major biological advance by providing molecular explanations of hypercholesterolemia. The plasma LDL level is the major determinant of the risk of vascular disease. We analyzed, also, C reactive protein (CRP), a cardiovascular risk factors coded by genes lying on Chromosome 21. Flow cytometric methods for measurement of LDL receptor on peripheral blood mononuclear cells (PBMC) may be used to identify patients with familial hypercholesterolemia [8]. Data in uremic patients suggest that a defect in LDL receptor function in PBMC may be due to a decrease in LDL receptor expression, which could contribute to the aberrant lipoprotein metabolism [9].

We therefore investigated LDL receptor expression on uninduced PBMC, particularly T lymphocytes because they express more LDL receptors than monocytes [10]. Since the progression of atherosclerosis is age-dependent, LDL receptor interactions are important in lipid plaque formation and T cells are present in early atherosclerotic lesions, interacting with LDL through the LDL receptor [11], we studied LDL expression on T lymphocytes in a group of old patients with DS.

## Methods

Blood samples were drawn from 19 old DS patients (male, average age 55 years) and 23 healthy individuals (male, average age 55 years) without dyslipidemia or any family history of coronary heart disease, no smokers or drunkers, with a Body Mass Index (BMI) < 25. Lipid measurements are given in Table 1. Plasma C reactive protein (CRP) concentration form DS and control was evaluated by LANIA (Latex Agglutination Nephelometric Immunoassay) technique (Biolatex, Spain). Samples were diluted 1:36 and results were calculated automatically by IMMAGE system. The minimum detectable concentration was 0.4 mg/dl.

**Table 1:** Cholesterol fractions in old patients with Down syndrome and healthy subjects. Means ± SD.

	**Healthy subjects**	**Down syndrome**
**Total cholesterol**	150 ± 19.64	152 ± 28.79
**Triglycerides**	55.9 ± 21.46	104.5 ± 50.2
**HDL-cholesterol**	48.4 ± 10.5	40.6 ± 4.24
**LDL-cholesterol**	88.3 ± 17.2	89 ± 24.4

None had been treated with lipid-lowering drugs before blood sampling. This study was conducted in accordance with the Declaration of Helsinki, 1975, amended in 1983.

Blood, collected in tubes containing EDTA, was cooled to 20°C and diluted 1:1 with Hank's buffered saline solution (HBSS, Biochrome, Biospa, Milan, Italy). PBMCs were prepared under sterile conditions, using Ficoll-Hypaque (Pharmacia Biotech, Milan, Italy) and diluted blood was layered in a centrifuge tube and centrifuged for 40 min at 400 *g*, 20°C. The interface containing the PBMCs was isolated, and the cells were washed three times in HBSS and resuspended in RPMI-1640 (Biochrome, Biospa, Milan, Italy) with L-glutamine (290 mg/L), penicillin (100,000 U/L), streptomycin (100 mg/L) and 100 mL/L human lipoprotein-deficient serum (HLPDS) to a final concentration of 10^6 ^cells/mL.

Tissue culture flasks were placed in ice-water for 60 min in the dark to reduce cell adhesion. PBMCs were removed by flushing with ice-cold HBSS (4°C) and washed twice in ice-cold HBSS with 20 mL/L HLPDS. The cell number was adjusted to 0.3 × 10^6 ^cells/mL, and 100-μL aliquots of cell suspension were pipetted into polypropylene tubes and placed in ice-water. Cells were incubated with 1.5 μg of monoclonal mouse anti-human LDL receptor-specific antibody, clone C7 (Amersham Life Science, Milan, Italy), for 30 min in the dark at 4°C. After this the cells were washed twice in ice-cold HBSS with 20 mL/L HLPDS, and incubated with 3 μL of fluorescein isothiocyanate (FITC, Dako Cytomation, Milan, Italy) for 30 min in the dark at 4°C. Cells were then incubated with 1 μL of R-phycoerythrin (RPE)-conjugated monoclonal antibody CD3-RPE or IgG_1_isotype-RPE for T lymphocytes.

The flow cytometry measurements were done in a FACScan flow cytometer (Becton Dickinson, Milan, Italy) equipped with a 15 mW, 488 nm, air-cooled argon laser and linked to a computer with CellQuest software. Forward scatter (FSC) and side scatter (SSC) were adjusted to exclude debris and dead cells. FITC emission was measured at 530 nm (FL1) and RPE emission at 585 nm (FL2); compensation was set using FITC-conjugated C7 (C7 FITC)-labeled cells (FL2-FL1) and CD3-RPE-labeled cells (FL1-FL2).

Means were compared by the unpaired *t*-test or one-way analysis of variance (ANOVA). Data are presented as means ± SD. Differences were considered statistically significant at *p *< 0.05.

## Results

**Figure 1:**
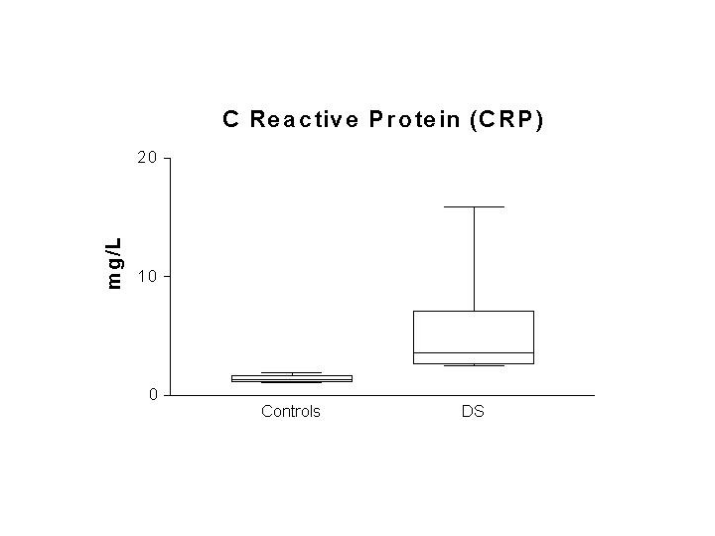
Levels of C reactive protein (CRP) in plasma from children with DS and age matched controls. Data are presented as mean ± S.D.

Table 1 shows cholesterol fractions of DS patients and healthy controls. DS total cholesterol and LDL did not differ from controls (*p *= 0.8 and *p *= 0.9 respectively). Blood levels of CRP were higher in DS than in controls, as illustrated in Figure 1(Controls 1.3 ± 0.3; DS = 5.7 ± 4.6 mg/L, p < 0.01). A regression analysis of data shows non relationship among CRP and cholesterol-related molecule levels.

**Figure 2:**
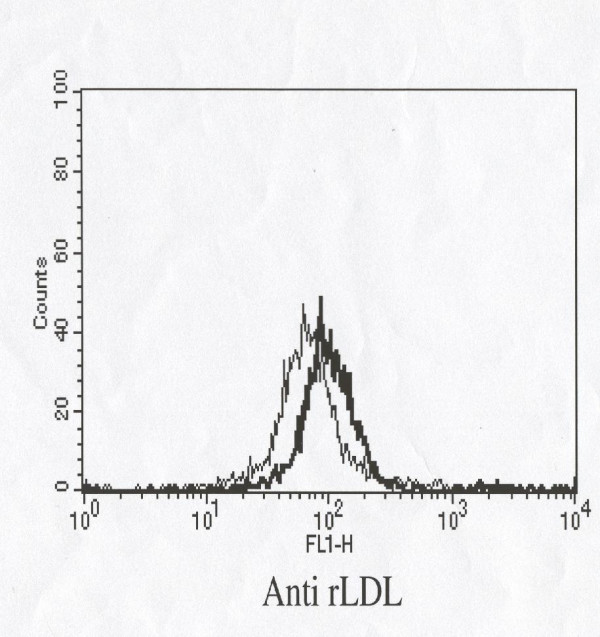
Mean fluorescence intensity (MFI) of LDL receptor expression in healthy subiects (bold line) and Down syndrome old subjects (fine line).

Triglycerides were higher, and HDL lower in DS patients (*p *< 0.01 and *p *< 0.05). Our data also show that the expression of LDL receptor on T lymphocytes was down-regulated in DS patients (Table 2 and Figure 2).

**Table 2:** Mean fluorescence intensity (MFI, %) of LDL receptor expression in healthy subjects and old patients with Down syndrome. Means ± SD.

	**Healthy subjects**	**Down syndrome**
**MFI (%)**	196.76 ± 20.54	139.87 ± 13.32

Mean intensity fluorescence (MIF) of LDL receptor expression was significantly different in DS patients and healthy controls (*p *< 0.0001) (Table 2).

## Discussion

Patients with DS who reach adolescence nowadays have a nearly normal life expectancy thanks to better medical care. When they die at a later age, cardiovascular diseases are less common than in the general population and they have even been proposed as "an atheroma-free model" [3]. Our results concerning cholesterol fractions suggest that DS patients should have a cardiovascular disease risk, if conclusions valid for the general population can be transferred to this category of patients. Although serum lipoprotein profiles cannot explain the lower prevalence of cardiovascular disease in individuals with DS, our triglyceride and HDL findings are in line with published figures. Similar findings were reported by other authors but in young patients [4,5].

Multiple factors are responsible for atherosclerosis, such as dietary habits but still the unexplained decline of LDL receptor expression with aging contributes importantly to borderline-high levels and cannot be ignored. For example the loss of estrogen-stimulated LDL receptor synthesis after menopause is an important contributor to elevated cholesterol in postmenopausal women. In addition, several genetic defects inherited singly appear to be causes of moderate hypercholesterolemia [10,12]. Generally defects of LDL receptor expression are associated with a high risk of premature atherosclerosis. In the elderly LDL receptor uptake is unexpectedly increased, and LDL receptor regulation and expression and serum LDL composition seem abnormal. There may also be alterations to the lipid metabolism of immune system cells during aging [13].

In our series, a reduction in LDL receptor expression was not correlated with high LDL serum levels or total cholesterol. Moreover, lipophospholipid (LPC) is generated by hydrolysis of phosphatidylcholine which is present in LDL; LPC may promote the start of an immune response and atherosclerosis may be the most extreme demonstration of this immune regulation pathway [14]. LPC may be a potent super-regulator of T-cell activation by inflammation at sites of tissue damage and in the early stages of atherosclerosis.

Interestingly, DS patients show no increase in their frequency of cardiovascular disease.

These conditions may be explained by mild immune defects in the syndrome, mainly involving macrophages and/or T_H_1 lymphocytes responses [15]. Alternatively, a over expression of atherosclerotic protective factors -yet unknown- maight be present in Down syndrome. As we reported earlier, the low incidence of cardiovascular disease in these patients and the high-risk factor of oxidatively modified LDL (oxLDL) [16] with – in this study – the low level of HDL, high levels of CRP and reduction of LDL receptor expression, lead to the conclusion that in this group of "healthy old" DS subjects, classical biochemical risk factors for atherosclerosis have been detected but risks, probably, are considerably lower.

## List of abbreviations

Down syndrome (DS); high-density lipoprotein (HDL); C reactive protein (CRP); Body Mass Index (BMI); low-density lipoprotein (LDL); peripheral blood mononuclear cells (PBMC); lipophospholipid (LPC).

## Competing interest

The author(s) declare that they have no competing interests.
